# Social Acceptance of Gas, Wind, and Solar Energies in the Canary Islands

**DOI:** 10.3390/ijerph18189672

**Published:** 2021-09-14

**Authors:** Rosario J. Marrero, Juan Andrés Hernández-Cabrera, Ascensión Fumero, Bernardo Hernández

**Affiliations:** 1 Department of Clinical Psychology, Psychobiology and Methodology, Universidad de La Laguna, 38205 Santa Cruz de Tenerife, Spain; jhernand@ull.edu.es (J.A.H.-C.); afumero@ull.edu.es (A.F.); 2 Department of Cognitive, Social and Organizational Psychology, Universidad de La Laguna, 38205 Santa Cruz de Tenerife, Spain; bhdezr@ull.edu.es

**Keywords:** renewable energy, fossil energy, risk perception, information, acceptance

## Abstract

Background: This study tested a theoretical model including key psychosocial factors that could be involved in the acceptance of different energy sources (gas, wind, and solar); Methods: Participants were 550 adult residents of the Canary Islands. Variables assessed were information and utility (normative motives), perceived risk and perceived benefits (gain motives), and negative and positive emotions (hedonic motives), with acceptance of each of the three energy sources as outcome variables; Results: It was found that renewable energies (wind and solar) had a higher degree of acceptance than non-renewable energy (gas). The proposed model satisfactorily explained the social acceptance of the three energy sources, although the psychosocial factors involved differed by energy source. The gain motives, mainly perceived benefits, were associated to a greater extent with gas energy, whereas normative motives, such as utility, and hedonic motives, such as positive emotions, had greater weight for renewables. Gender differences in gas energy were found. Information about renewable energy increased positive emotions and acceptance, whereas information about fossil fuel-based energy generated more negative emotions and perceived risk, decreasing acceptance; Conclusions: Utility, perceived benefits and positive emotions were involved on the acceptance of both renewables and non-renewables. The theoretical model tested seems to be useful for understanding the psychosocial functioning of the acceptance of the various energy sources as an essential aspect for the transition of non-renewable to renewable energies.

## 1. Introduction

In recent years, the development and application of secure and sustainable energies has become a great challenge [1,2]. In the Canary Islands, electricity consumption was 4121 kWh/inhabitants in 2019 [3]. In that same year, electricity generation with non-renewable energies constituted the main energy resource (83.6%), whereas renewable sources represented only 16.4% of the total [3]. However, the Canary Islands have numerous natural energy resources, such as wind and sun, from which the region could benefit. Research has been extensive on challenges at the political, technical, or financial level, but little analysis of public opinion about renewable energies [4]. This research aims to respond to a gap in the different psychosocial motives underlying the acceptance of the various sources of energy (renewable and non-renewable). Another gap has consisted in contrasting the theoretical model of acceptance based on gender. In addition, in geographically limited island territories, the potential environmental impact of different energy technologies is little known. Therefore, discovering the level of social acceptance and the psychosocial processes that can explain it is very important.

Acceptance refers to a favorable response related to a fact that is manifested in the form of opinion or support, consent, or authorization [5] and is a prerequisite for implementing new energy models. Acceptance includes both cognitive and affective dimensions and could be analyzed at country, local, or household level [6]. Research on the social acceptance of energy-related technologies has been developed from three approaches: one focused on the risk perception tradition, another focused on the psychosocial factors of community members and how to participate in the decision-making of politicians and technology developers, and a third focused on minority groups that are in a situation of discrimination or inequality in terms of use of or access to energy [7]. It is possible to investigate the acceptance of energy sources by incorporating variables related to risk perception and psychosocial processes, and by taking into consideration the environmental context.

Based on goal-framing theory [8], three kinds of motives could be involved in the social acceptance of the various energy sources (gas, wind, and solar): normative motives that imply acting in a socially or morally appropriate way, e.g., evaluating the positive or negative effects of an action on the environment, assessed in this study by means of utility and information; gain motives related to personal resources and resource efficiency, e.g., evaluating the costs and benefits of an action, assessed through perceived risk and perceived benefits; and hedonic motives associated with what makes the person feel better, e.g., avoiding negative feelings or seeking pleasure, in this study measured through positive and negative emotions.

Normative motives, such as utility and information of energy sources, can affect acceptance of energy sources in different ways. Utility is associated with the perceived value of an action or situation and individuals usually decide based on perceived value [9]. Utility may have a direct relationship with the acceptance of energy sources since it implies a cognitive assessment of the gains or losses of a certain performance. It could also indirectly affect acceptance through emotions. On the other hand, information has proven to be a key element in the acceptance of energy sources. Information can have positive or negative effects on acceptance, depending on the type of energy considered [10,11]. Providing information about undergrounding high-voltage power lines decreases acceptance, perceived benefits, and positive feelings and increases perceived risk [12]. However, people with information about renewable energy accept this type of energy to a greater extent and are willing to pay for it [13].

Gain motives, such as perceived risk and perceived benefits, have been extensively studied. People engage in certain behaviors after estimating the potential gain or benefits and the costs or risk involved, selecting the behavior that brings the greatest reward to them or to society as a whole [14]. Perceived risk is negatively associated with acceptance of energy [15]. Risks can include financial, security, health, or environmental risks [16]. For example, risk associated with the cost of renewable energy technology reduces acceptance [11]. Perceived benefits are positively associated with acceptance of different technologies [17]. Benefits include cost savings, job creation, energy efficiency, or environmental friendliness [16,18].

Hedonic motives are associated with the emotions that are generated by any stimulus or situation that the individual faces [19]. Emotions have been shown to be involved in decision-making and play an important role in opposing or accepting energy projects [20,21]. Emotions could function as a marker that precedes the perception of risks and benefits, which does not imply that the assessment is irrational but that the person includes a moral connotation to his/her assessment [22]. The emotional evaluation that takes place before a project is launched may affect the acceptance of that project [20,23].

Few studies compare acceptance models including more than one type of energy simultaneously. In this sense, people show higher acceptance of solar and hydro power compared to wind, gas-fired, and nuclear power [24,25]. Additionally, perceived benefits, trust, and positive emotions have been found to be relevant predictors of acceptance in five types of energy (solar, hydro, wind, nuclear, and gas-fired), whereas negative emotions and perceived cost decrease acceptance [26].

On the other hand, some studies have found gender differences in the perception of energy sources and technologies [27,28]. Men report higher acceptance of hydroelectric energy and women of solar energy [26]. Other studies have found that women perceive more risk associated with waste-to-energy facilities [29], but no gender differences are found in perceived benefits of gas-fired power plants [26]. Considering the mixed results for gender differences in the acceptance of different energy sources, it is necessary to analyze the impact of gender.

The purpose of this study was to test the goal-framing theory including key psychosocial factors (information, utility, positive and negative emotions, and perceived risk and benefits) that could be involved in acceptance of different energy sources (gas, wind, and solar) in an extremely remote community, such as that of the Canary Islands.

## 2. Materials and Methods

### 2.1. Participants

Participants were a convenience sample of 550 adult residents in the Canary Islands. A total of 58.2% were women and 41.8% were men ranging in age from 17 to 83 (M = 34.86, *S.D.* = 16.78). Of the total sample, 3.1% had no formal studies, 10.4% had a primary education, 35.8% had a secondary education, and 50.7% had a university education. Half of the sample had a low socioeconomic level (50.9%); a third had a medium socioeconomic level (33.3%), and a few had a high socioeconomic level (15.8%). Distributions of sociodemographic variables were compared with data from the Spanish population. Our sample was similar in gender, where national figures show 50.98% of women versus 49.02% of men [30], and socioeconomic level (income), with 47.22% of the Spanish population having a low income, 31.44% a medium income, and 21.54% a high income [31]. In education, our sample showed a higher educational level compared to the national distribution, which is around 39.9% for primary education, 22.85% for secondary education, and 37.25% for university education [32]. In age, our sample was younger than the national mean, which lies at 43.38 years [30].

### 2.2. Instruments

A checklist was used to record sociodemographic characteristics, such as age, gender, educational level (no formal studies, primary education, secondary education, and university education), and socioeconomic level (monthly income below EUR 1500, between EUR 1501 and 2500, over EUR 3001). Appendix A shows the assessment protocol with the questions designed for this research. All variables were measured through a 5-point Likert scale (1 = strongly disagree, 5 = strongly agree):

1. Information assessed, through seven items, whether individuals perceived that they were sufficiently informed about the different types of energy; what the source of information was (authorities, experts, or media); and if they knew the effects of energy sources on health. Cronbach’s alpha for the entire scale was 0.82 for gas, 0.79 for solar, and 0.78 for wind.
2. Utility assessed, through two items, the extent to which each of these energy sources could be efficient and would meet the needs of the individual. Cronbach’s alpha for the entire scale was 0.81 for gas, 0.78 for solar, and 0.76 for wind. Only one item was considered in the model since the correlations between the two items were high, above 0.60.
3. Perceived benefits. Participants were given a list of ten items that assess perceived benefits of different sources of energy, such as economic development, reduced unemployment, sustainability, and improvements in the standard of living of the local community. Cronbach’s alpha for the entire scale was 0.93 for gas, 0.91 for solar, and 0.90 for wind.
4. Perceived risk was assessed through five items about the risk from different energy sources to the environment, to health, to the economy, or of serious accidents. Cronbach’s alpha for the entire scale was 0.86 for gas, 0.87 for solar, and 0.85 for wind.
5. Positive and negative emotions were assessed through ten positive adjectives and ten negative adjectives from the Positive and Negative Affect Schedule (PANAS) [33]. In this study, Cronbach’s alpha for the negative emotions scale was 0.94 for gas, 0.91 for solar, and 0.91 for wind. Cronbach’s alpha for the positive emotions scale was 0.80 for gas, 0.86 for solar, and 0.87 for wind.
6. Acceptance assessed, through four items, the extent to which the individual agrees with and supports the idea that these types of energy can be used or fostered. All items were included in the measurement model. Cronbach’s alpha was 0.90 for gas, 0.82 for solar, and 0.78 for wind.

### 2.3. Procedure

The participants were recruited through Psychology students from the University of La Laguna. The students, who received academic credit for their participation in the research, administered the assessment protocol, which was hosted on a web link, via email to at least three people from their social environment. The students were asked to ensure that the three people covered a specific age and gender quota to increase sample variability. Participants gave informed consent to their answers being used for research purposes by pressing the send key after having filled out the form. The questionnaires took about 35 min to complete. At the end, a contact telephone number was requested for data-checking purposes. A data check was carried out on 10% of participants randomly distributed among the interviewers. No incongruences were identified, and data collection was considered valid; no participant was removed through this procedure. The study was conducted in accordance with the Declaration of Helsinki, and the ethical approval for the conduct of the research study was granted by the Animal Welfare and Research Ethics Committee of the University of La Laguna.

### 2.4. Statistical Analyses

**Figure 1:**
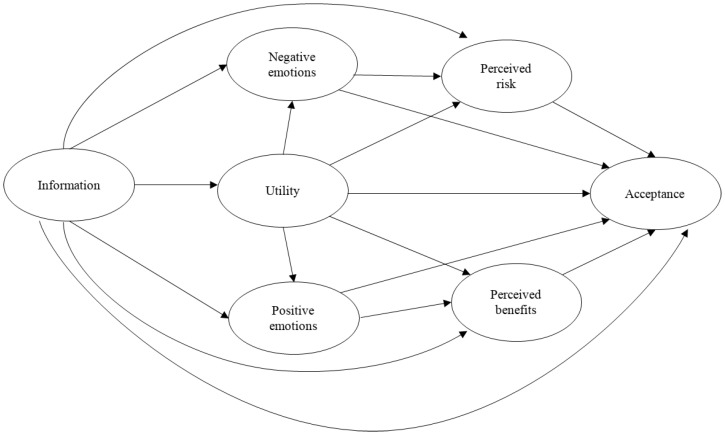
Proposed theoretical model.

First, mean differences between the acceptance of the three energy sources were applied. Second, Pearson correlational analyses were used to study the relationships between the variables included in the study. Then, Structural Equation Modeling (SEM) with latent variables was performed [34] to test the proposed models, using R library lavaan [35] with ULLRToolbox [36]. Latent variables were constructed using the four most representative items for each measurement scale (information, perceived risk and benefits, positive and negative emotions, and acceptance), except for utility, where only one item was used. The measurement models with the most representative items were first tested to assess the extent to which the latent variables were well represented by the observed indicators of each structural model. Then, the proposed structural model (Baseline Model) was tested for each of the energy sources (Figure 1). In this model, information, perceived risk and benefits, and positive and negative emotions were included as antecedent variables, and acceptance as an outcome variable. Analyses of covariance structure were performed for the entire sample. The structural model invariance by gender was tested through multigroup SEM estimation. First, a configural model was tested with two groups (male and female) without any constraints. Second, a measured model was estimated where the factor loadings were constrained to be the same for both groups (male and female). In a next step, a structural model was examined where factor loading and regression parameters were constrained to be the same for both groups (male and female). The structural differences between both groups were examined comparing the configural model with the constrained structural model. The statistics used for testing the acceptability of the models were: χ2, goodness-of-fit index (GFI), comparative fit index (CFI), Tucker–Lewis index (TLI), normative fit index (NFI), non-normed fit index (NNFI), standardized root mean square residual (SRMR), and the root mean square error of approximation (RMSEA). The expected values for an acceptable fit were around 0.90 for the CFI, TLI, GFI, NFI, and NNFI indexes [37]. The well-fitting models obtained SRMR values under 0.05, and RMSEA values of 0.08 indicated a reasonable fit of the model [38]. A confidence interval of 90% was established.

## 3. Results

### 3.1. Descriptive and Correlational Analysis

Table 1 presents means and standard deviations of information, utility, perceived risk and benefits, positive and negative emotions, and acceptance measures for each energy source. A comparison of the means indicated that solar energy was more accepted than gas and wind energy (contrast between solar and gas: F(1549) = 1261.23, *p* < 0.001, η2 = 0.70; contrast between solar and wind: F(1549) = 90.99, *p* < 0.001, η2 = 0.14). Additionally, significant differences appeared between wind and gas energy, showing more acceptance for wind: F(1549) = 971.72, *p* < 0.001, η2 = 0.64. Renewable energies had a higher degree of acceptance than non-renewables, in line with other studies that report a 90% support for renewable energy [39,40].

Pearson correlations were obtained between acceptance and all other variables for each energy source (Table 2). The acceptance of different energy sources was positively related to utility, perceived benefits, and positive emotions, whereas it was negatively related to perceived risk and negative emotions. Previous research confirms the relationships found between psychosocial processes and acceptance of an oil drilling project [41]. Utility and perceived benefits were the variables most associated with acceptance of different energy sources. The literature supports a greater relationship of acceptance with the perceived benefits than with the perceived risks [26]. Renewable energies (solar and wind) acceptance showed a smaller correlation with negative emotions than gas energy. The adverse effects on the environment that non-renewable energies have already shown are likely to be influencing their negative evaluation [13]. Information was positively associated with the acceptance of renewable energies but was barely related to gas energy acceptance. Other studies have found that information about renewable energy increases acceptance to pay for it [13].

### 3.2. Structural Equation Modeling (SEM)

**Figure 2:**
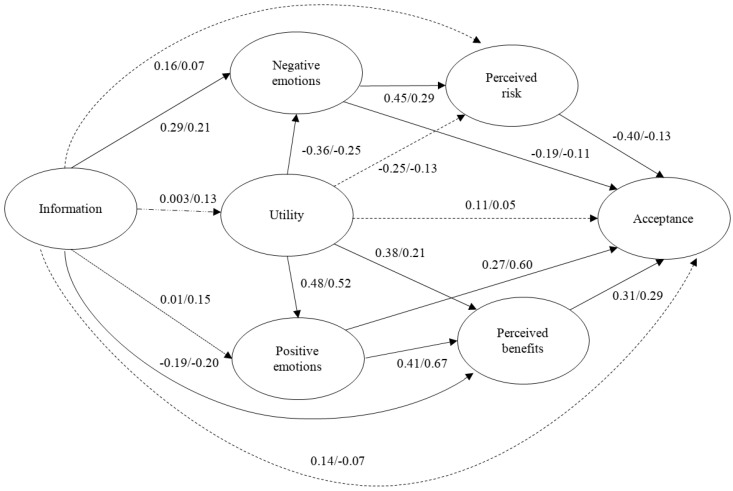
Structural model obtained for gas energy acceptance. The first beta value is for females and the one that follows the slash (/) is for males. The dotted lines show non-significant paths for females. The dashed lines show non-significant paths for males. The dotted and dashed lines show non-significant paths for both females and males.

First, the measurement model was tested for seven latent constructs in each energy source. The factor loadings for the indicators on the latent variables indicated that the latent constructs were well represented by their indicators. The proposed model in Figure 1 was checked through SEM for each energy source. The theoretical model showed a good fit to the data for gas energy: χ2 (258, N = 550) = 686.93, *p* < 0.001; CFI = 0.95; TLI = 0.94; NFI = 0.92; NNFI = 0.94; SRMR = 0.09; RMSEA = 0.055, 90% CI = (0.05, 0.06) (Figure 2). The covariance of the measurement errors of items 3 and 4 of acceptance had to be released (permit values not equal to 0) to achieve the model fit. Most of the hypothesized relationships were significant. Gain (perceived benefits and risk) and hedonic (positive and negative emotions) motives explained acceptance of gas energy to a greater extent. It seems that, when making a judgment about an energy source, people combine both rational processes related to the perception of risk and benefits, as well as more emotional elements, whether positive or negative [19]. The variables included in the model explained 78.2% of the variance of gas energy acceptance. This model was then used with multigroup estimation to check whether the model was the same for males and females. The configural model showed adequate fit indexes: χ2 (516, N = 550) = 1004.71, *p* < 0.001; RMSEA = 0.059, 90% CI = (0.053, 0.064); NFI = 0.89; NNFI = 0.93; CFI = 0.94. Next, the measured model with constrained factor loadings for both groups was tested and continued to show an adequate fit: χ2 (534, N = 550) = 1022.96, *p* < 0.001; RMSEA = 0.058, 90% CI = (0.052, 0.063); NFI = 0.89; NNFI = 0.94; CFI = 0.94. Comparison between both configural and metric models, through chi-squares differences, indicated that the two models were indistinguishable (χ 2diff = 18.25, *p* = 0.439), so the invariance of measure was demonstrated for men and women. The structural model with constrained loading and regression for both genders also showed an adequate fit: χ2 (551, N = 550) =1080.92, *p* < 0.001; RMSEA = 0.059, 90% CI = (0.054, 0.064); NFI = 0.88; NNFI = 0.93; CFI = 0.94. Significant differences between this structural model and the configural model were found (χ 2diff = 76.21, *p* < 0.001), indicating significant differences between males and females in all structural parameters. These differences are shown in Figure 2, a slash (/) between values indicating the differences between coefficients for males and females. The paths between information and perceived risk, as well as utility and information over acceptance, were significant for females but not for males. Previous research has found that females show a greater perception of danger [42]. Information had a significant effect over positive emotions for males but not for females. This result could be explained because males employ more approximate strategies focused on achievement, which could lead to a greater experimentation of positive emotions [43]. The model accounted for adequate proportions of variance in gas energy acceptance for both males (82.4%) and females (79.4%).

**Figure 3:**
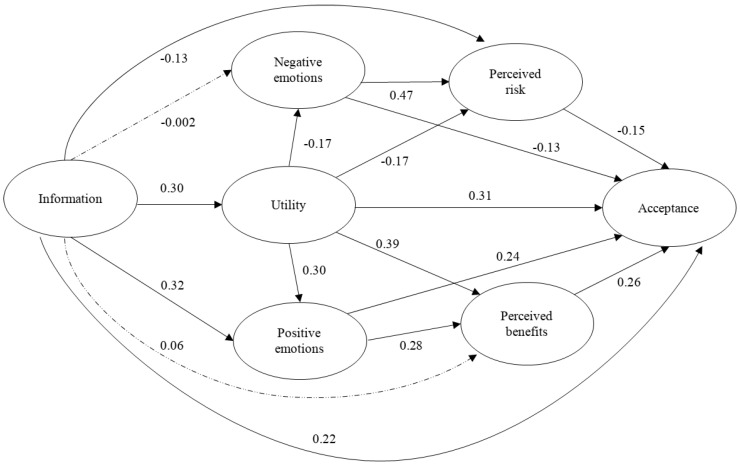
Structural model obtained for solar energy acceptance. The dotted and dashed lines show non-significant paths.

The theoretical model was tested for solar energy (Figure 3). Again, an adequate fit of the model to the data was found: χ2 (257, N = 550) = 579.07, *p* < 0.001; CFI = 0.95; TLI = 0.94; NFI = 0.91; NNFI = 0.94; SRMR = 0.06; RMSEA = 0.048, 90% CI = (0.043, 0.053). The covariance of the measurement errors of items 3 and 4 of acceptance, as well as 1 and 2, had to be released (permit values not equal to 0) to achieve the model fit. Information showed a significant relation with utility, positive emotions, perceived risk, and acceptance. As noted above, information about renewable energies seems to influence not only a greater acceptance of them [13], but also of other psychological processes related to a positive assessment, and uncovers the possible risks of this energy source. Utility also had a strong relationship with positive emotions, perceived benefits, and acceptance. The environment in which the research was carried out, characterized by a warm climate, may have influenced this more positive perception of the utility of solar energy, thus promoting acceptance. The impact of solar energy has been universally positive [44]. In this line, negative emotions were also found to make an important contribution to perceived risk but a lower contribution to acceptance, whereas positive emotions had a greater weight in acceptance. Acceptance was mainly explained by utility, perceived benefits, and positive emotions. The variables included in the model explained 79.6% of the variance of solar energy acceptance. When the multigroup estimation was tested for males and females, an adequate fit for the configural model was found: χ2 (514, N = 550) = 948.91, *p* < 0.001; RMSEA = 0.055, 90% CI = (0.05, 0.061); NFI = 0.87; NNFI = 0.92; CFI = 0.93. Next, the measured model with constrained factor loadings for both groups was tested and still showed an adequate fit: χ2 (532, N = 550) = 969.76, *p* < 0.001; RMSEA = 0.055, 90% CI = (0.049, 0.06); NFI = 0.86; NNFI = 0.93; CFI = 0.93. Comparison between both configural and metric models indicated that the two models were indistinguishable (χ 2diff = 20.85, *p* = 0.287), proving the measurement invariance. The structural model with constrained loading and regression for both genders also showed an adequate fit: χ2 (549, N = 550) = 984.81, *p* < 0.001; RMSEA = 0.054, 90% CI = (0.048, 0.059); NFI = 0.86; NNFI = 0.93; CFI = 0.93. The differences between this structural model and the configural model were non-significant (χ 2diff = 35.90, *p* = 0.426). The model accounted for adequate proportions of variance in solar energy acceptance for both males (86.4%) and females (73.4%).

**Figure 4:**
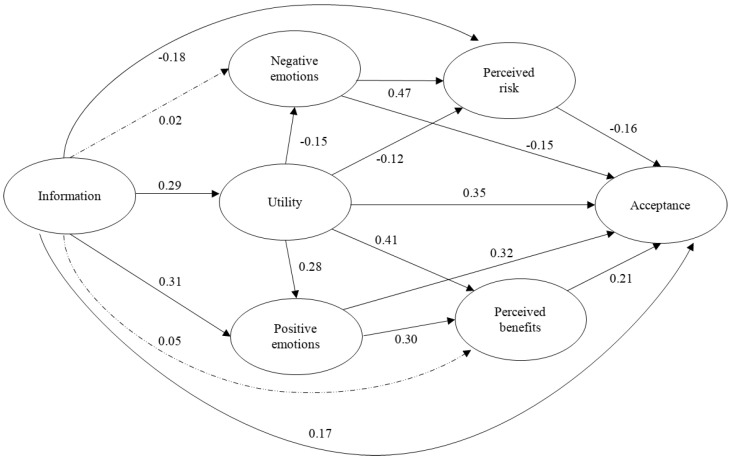
Structural model obtained for wind energy acceptance. The dotted and dashed lines show non-significant paths.

The proposed model also showed a good fit to the data for wind energy (Figure 4): χ2 (257, N = 550) = 607.75, *p* < 0.001; CFI = 0.94; TLI = 0.93; NFI = 0.90; NNFI = 0.93; SRMR = 0.06; RMSEA = 0.05, 90% CI = (0.045, 0.055). Here, also the covariance of the measurement errors of items 3 and 4 of acceptance, as well as 1 and 2, had to be released (permit values not equal to 0) to achieve the model fit. In the same way as for solar energy acceptance, information was a significant predictor of utility, positive emotions, perceived risk, and acceptance. The contribution of utility to the variance of acceptance was both direct and indirect, mainly through perceived benefits. Again, the results coincided with those obtained for the acceptance of solar energy. Similarly, negative emotions showed a significant relationship with perceived risk but the contribution to acceptance was low. Positive emotions explained acceptance to a greater extent, both directly and through perceived benefits. The results indicated that the psychological processes involved in the acceptance of both types of renewable energy (solar and wind) were similar, coinciding with previous research that emphasizes the high level of support for these energy sources [40]. The variables included in the model explained 81% of the variance of wind energy acceptance. When the multigroup estimation was tested for males and females, an adequate fit for the configural model was found: χ2 (514, N = 550) = 1015.47, *p* < 0.001; RMSEA = 0.06, 90% CI = (0.054, 0.065); NFI = 0.85; NNFI = 0.90; CFI = 0.92. Next, the measured model with constrained factor loadings for both groups was tested and also showed an adequate fit: χ2 (532, N = 550) = 1031.37, *p* < 0.001; RMSEA = 0.058, 90% CI = (0.053, 0.064); NFI = 0.85; NNFI = 0.91; CFI = 0.92. Comparison between both configural and metric models indicated that the two models were indistinguishable (χ 2diff = 15.90, *p* = 0.599), and the measurement invariance was tested through gender. The structural model with constrained loading and regression for both genders also showed an adequate fit: χ2 (549, N = 550) = 1050.53, *p* < 0.001; RMSEA = 0.058, 90% CI = (0.052, 0.063); NFI = 0.84; NNFI = 0.91; CFI = 0.92. The differences between this structural model and the configural model were non-significant (χ 2diff = 35.06, *p* = 0.465). The model accounted for adequate proportions of variance in wind energy acceptance for both males (82.2%) and females (77.6%).

## 4. Discussion

The present research tested a conceptual model to examine social acceptance of renewable (solar and wind) versus non-renewable (gas) energies in the Canary Islands. The results indicated that information, utility, perceived benefit, perceived risk, and emotions were significantly associated with acceptance of the three energy sources, although the relative importance of the different psychosocial factors varied depending on the energy source.

As expected, renewable energies, mainly solar energy, had a higher degree of acceptance than non-renewable energy. Previous research has found that support for renewable energy is around 90% and is stronger than support for fossil fuel-based energy sources or nuclear energy [39,40]. The current study seems to show that people evaluate different energy sources by comparing them against each other rather than evaluating them in isolation. When renewable and non-renewable energies are simultaneously evaluated, people′s preferences lean toward renewables, although some renewable infrastructures entail a certain risk [45]. Solar energy has turned out to be the preferred choice of the participants in this study, in line with other studies [46]. Available worldwide, it is one of the best substitutes for fossil energy [4]. The greater acceptance of solar versus wind energy is probably because of a more positive perception of the sun than of wind [44]. Tolerance to warm temperatures has been found to be higher than to cool temperatures, and a low wind speed is more agreeable than a high wind speed [47]. Regarding the possible disadvantage of renewable energy, although both solar and wind energy occupy large areas of land, wind energy has more impact on the landscape than solar energy and entails the construction of high-voltage lines to conduct the maximum amount of electricity generated [48].

For renewable energy, utility, perceived benefit, and positive emotions were the most powerful predictors of acceptance. Utility had both direct and indirect relationships on acceptance, through perceived benefits (gain motives). Year-round warm weather and the prevailing trade winds in the Canary Islands may be influencing the perception of the greater utility of renewable energy versus gas energy. Moreover, the pathways through positive emotions and perceived benefits had a higher weight on acceptance of renewable energy than the pathways through negative emotions and perceived risk. Affect has been shown to influence the formation of attitudes toward wind energy projects; mainly opponents tend to show contradictory feelings [49]. Renewable energy comes from natural resources and is considered clean and environmentally friendly. It emerges as technically and economically viable, as well as socially accepted [13]. Hence, a priori, it can generate positive emotions and a greater perception of benefits in the population. In fact, perceived benefits had a greater impact on acceptance than perceived risks, which was consistent with previous studies [17,26]. Information had direct effects of low magnitude on acceptance but played an important role through positive emotions and utility. Information seems to be more associated with personal experience and emotions than with acceptance of renewable energy [50]. However, empirically supported information is necessary to build trust and promote a positive attitude in the population toward renewable energies [13]. Some studies about support for wind energy suggest that participating in informational events increases initial support, mainly for people with lower initial confidence [51]. In general, the population is in favor of a transition to renewable energy, but the technology required for implementation is accepted when located far from homes [52].

Regarding gas energy, the results indicated that utility showed only indirect effects, through positive emotions, negative emotions, and perceived benefits, over acceptance for both men and women. It seems that when the person perceives the utility of gas energy, contradictory emotions arise. However, the results showed that positive emotions and the perception of benefits eventually prevail, which leads to the acceptance of this energy source. Previous studies suggest that perceived benefits explain public acceptability of nuclear and gas energy [53]. However, gender differences appeared in different psychosocial processes involving acceptance: females showed a greater focus on perceived risk and negative emotions, and males paid greater attention to positive emotions. The differences between men and women are consistent with some studies that indicate that women are more oriented to avoiding threat and men to achievement [43]. Several studies have found that males show a higher exploration of environment for resources and seek new experiences even though they are risky, whereas female sex hormones could inhibit exploratory behavior, influencing a greater perception of danger and risks in the environment [42]. Perceived risk has also been found to be greater for people less familiar with that energy source [53]. Gender differences did not appear in renewable energy, perhaps because, in general, it is perceived to have few risks and high benefits. Renewable energy has emerged precisely to put an end to the risks and other disadvantages of non-renewable energies. That there is a greater need to implement them is therefore accepted, and they appear to be more linked to a democratic and egalitarian organization of power [54]. People who are committed to the common good and who value equality show greater acceptance of solar and wind energy technologies in their neighborhood [55].

Although the findings of this study allow us to establish the adequacy of the proposed model, it is not free of limitations. First, the sample, while relatively wide and diverse, was a convenience sample in which young people with university studies predominated. Second, this study does not allow us to identify differences in the function of other contextual variables, such as area of residence, or economic and environmental impact. For example, support for renewable energies has been observed at a general level, but more negative attitudes are found among residents [11,56]. Third, there was no information about which governmental or private entity was proposing one type of energy or another. Greater support was given when the technological development was carried out by the local government compared with a private developer [57]. Finally, the causal order of the variables in the proposed model are discussed. Some studies indicate that cognitions (perceived risks and benefits) precede emotions [58]. However, in this study, people′s judgments were considered to be based on their feelings [23,46]. Emotions were therefore considered antecedent variables of cognition.

Despite these limitations, the proposed model allows us to integrate several factors involved in acceptance into a conceptual framework where gain motives showed a close relationship with acceptance of gas energy, whereas normative motives were more relevant for renewable energy. Hedonic motives played an important role in the acceptance of both renewable and non-renewable energy sources. In this study, different socio-psychological factors embedded in the goal-framing theory were considered as concomitant to explain the acceptance of renewables and non-renewables energies. It has been suggested that positive affective reactions toward renewable energy are necessary, though they may be insufficient, if acceptance is to be achieved. Providing appropriate information about the utility and benefits of renewable energy could also help accelerate the application of renewable energy projects. Another important practical implication could consist in introducing emotional education from the early stages of development at elementary school, since emotional elements involved in acceptance have been identified.

The research findings add to our understating of social acceptance of energy sources and provide important implications for promoting the transition from fossil fuel-based energy to renewable energy. In future, in addition to acceptance, other responses such as support, tolerance, and even indifference could be studied. Another clearly relevant area for future empirical development are the dynamics of people’s responses to renewables over time, at local, national, and global levels. In this sense, it would be interesting to conduct research from a multi-level perspective that would simultaneously examine the impact of the use of renewables at different levels and how the way in which different stakeholders function affects the perception of individuals locally. The Canary Islands have a year-round subtropical climate. The warm temperatures and trade winds are therefore ideal for investing in renewable energy. If attention is paid at government level to what is needed to reside in the islands, it may be possible to create a fairer and more sustainable energy model based on the region′s natural resources.

## 5. Conclusions

In this study, renewable energies were more widely accepted than non-renewable energy. Solar energy, in particular, was the most widely accepted energy source. Utility, perceived benefits and positive emotions were the psychosocial processes most involved in the acceptance of different energy sources, whether renewable or non-renewable.

The theoretical model tested seems to be useful for understanding the psychosocial functioning of the acceptance of the various energy sources as an essential aspect for the transition of non-renewable to renewable energies. Gain motives, mainly perceived benefits, were more important in non-renewable energy acceptance, although gender differences were found, whereas normative (utility) and hedonic motives (positive emotions) carried more weight for the acceptance of renewable energies. Information about renewable energy increased positive emotions and acceptance, whereas information about non-renewable energy generated more negative emotions and perceived risk, decreasing acceptance.

The integration of the three approaches to risk perception, the psychosocial factors involved in decision-making, and egalitarian access to energy resources could provide an answer to the problem of acceptance and implementation of renewable energies in future. In this sense, starting from theoretical models tested on acceptance could be a first step toward the design and development of campaigns focusing on the utility, benefits, and emotions generated by renewable energy sources. In this process, of special importance is community participation, considering the idiosyncrasies and establishment of democratic relations between the agents involved and the members of the community. In future, knowing people′s opinions in real time, through social media, could be a useful strategy for policymakers and experts to design and disseminate campaigns aimed at promoting renewable energies. Nevertheless, adopting renewables in a mixed model could be more widely accepted by the community and could contribute substantially to the objectives of environmental and socioeconomic policies.

## Figures and Tables

**Table 1:** Means and standard deviations of measured variables for each energy source.

	Gas Energy		Solar Energy		Wind Energy	
	Mean	S.D.	Mean	S.D.	Mean	S.D.
Information	2.48	0.85	2.93	0.84	2.92	0.83
Utility	3.04	1.18	4.24	0.86	4.12	0.92
Positive emotions	2.43	0.76	3.36	0.90	3.31	0.92
Negative emotions	2.87	1.14	1.80	0.84	1.84	0.85
Perceived risk	3.28	1.07	1.73	0.91	1.79	0.92
Perceived benefits	2.77	1.04	4.33	0.73	4.33	0.72
Acceptance	2.26	1.11	4.40	0.83	4.16	0.88

**Table 2:** Pearson correlations between antecedent variables and acceptance for each energy source.

	Gas Energy Acceptance	Solar Energy Acceptance	Wind Energy Acceptance
Information	0.05	0.34 ***	0.30 ***
Utility	0.56 ***	0.60 ***	0.56 ***
Positive emotions	0.43 ***	0.43 ***	0.44 ***
Negative emotions	−0.44 ***	−0.23 ***	−0.20 ***
Perceived risk	−0.56 ***	−0.33 ***	−0.29 ***
Perceived benefits	0.73 ***	0.54 ***	0.49 ***

Note: *** *p* < 0.001.

## Data Availability

The data presented in this study are openly available in https://riull.ull.es/xmlui/handle/915/24874 (accessed on 21 July 2021).

## Appendix A

Information

1. To what extent do you consider yourself informed about this type of energy?
2. To what extent do you have information from public authorities on this type of energy?
3. To what extent do you have information on this type of energy from experts or specialized documents?
4. To what extent do you have information from the media about this type of energy?
5. When you hear, see, or read something about this type of energy, do you seek as much information as possible?
6. Do you know the mechanisms required to generate this type of energy?
7. Do you know the effects of this energy source on people′s health?

Utility

1. To what extent do you think this type of energy will meet your needs?
2. To what extent do you think this type of energy can be efficient?

Perceived benefits

1. Promoting this type of energy will increase the standard of living of people who live in the Canary Islands.
2. The prestige of the Canary Islands will increase by promoting this type of energy.
3. Enhancing this type of energy will provide economic benefits to the Canary Islands.
4. More investment will come to the islands by promoting this type of energy.
5. The exploitation of this type of energy will help to overcome the economic crisis in the Canary Islands.
6. Enhancing this type of energy will help create new jobs in the Canary Islands.
7. Encouraging this type of energy will contribute to the social progress of the Canary Islands.
8. Increasing this type of energy will save money.
9. This type of energy respects the environment.
10. Enhancing this type of energy is compatible with tourism development.

Perceived risks

1. This type of energy involves a risk to the environment.
2. This type of energy involves a risk to people′s health.
3. This type of energy involves a risk to the economy of the Canary Islands.
4. In general, this type of energy poses a risk to the society of the Canary Islands as a whole.
5. To what extent do you consider that this type of energy can cause a serious accident?

Acceptance

1. In general, you accept this type of energy.
2. In general, you are in favor of promoting this type of energy.
3. You would support the promotion of the installation of this technology near your place of residence.
4. You would accept the installation of the devices required for the operation of this technology near your place of residence.
